# Institutionalizing the Management of Sick Young Infants: Kenya’s Experience in Revising National Guidelines on Integrated Management of Newborn and Childhood Illnesses

**DOI:** 10.9745/GHSP-D-22-00482

**Published:** 2023-04-28

**Authors:** Wilson Liambila, Fred Were, Timothy Abuya, George Odwe, Alice Natecho, Samuel Mungai, Peter Mwaura, David Githanga, Joe Mbuthia, Doris Kinuthia, Allan Govoga, Charlotte E. Warren, Kezia K’Oduol, Jesse Gitaka

**Affiliations:** a Population Council, Nairobi, Kenya.; b Kenya Paediatric Research Consortium, Nairobi, Kenya.; c Fountain Africa Trust CBO, Webuye, Kenya.; d Centre for Research in Infectious Diseases, Directorate of Research and Innovation, Mount Kenya University, Thika, Kenya.; e Division of Neonatal and Child Health, Ministry of Health, Nairobi, Kenya.; f Population Council, Washington, DC, USA.; g Living Goods, Nairobi, Kenya.

## Abstract

**Introduction:**

In 2015, the World Health Organization (WHO) developed guidelines for the management of sick young infants (SYIs) with possible serious bacterial infection (PSBI) where referral is not feasible. The Ponya Mtoto project was designed as an implementation research project to demonstrate how to adopt the WHO PSBI guidelines in the Kenyan context.

**Ponya Mtoto Project Description:**

Between October 2017 and June 2021, Ponya Mtoto was implemented in 4 Kenyan counties with higher infant and newborn mortality rates than the national mean. A total of 48 health facilities stratified by level of services were selected as study sites.

**Implementation Approach:**

The following activities were done to institutionalize the management of SYIs with PSBI where referral is not feasible in Kenya’s health system: (1) participating in a cocreation workshop and development of a theory of change; (2) revising the national integrated management of newborn and childhood illnesses guidelines to incorporate the management of PSBI where referral is not feasible; (3) improving availability of essential commodities; (4) strengthening provider confidence in the management of SYIs; (5) strengthening awareness about PSBI services for SYIs at the community level; and (6) harmonizing the national integrated management of newborn and childhood illnesses guidelines to address discrepancies in the content on the management of PSBI. In addition, the project focused on strengthening quality of care for SYIs and using implementation research to track progress in achieving project targets and outcomes.

**Conclusion:**

Using an implementation research approach to introduce new WHO guidelines on PSBI where referral is not feasible into Kenya’s health care service was critical to fostering engagement of a diverse range of stakeholders, monitoring provider skills and confidence-building, strengthening provision of key commodities for managing SYIs with PSBI, and sustaining community-facility linkages.

## Abstract (precis)

Using implementation research to introduce guidelines on management of sick young infants with possible severe bacterial infection into Kenya’s integrated management of newborn and childhood illness guidelines improved uptake of services for sick young infants.

## Teaser

**Key Findings:**

Key steps in institutionalizing guidelines for possible severe bacterial infection into Kenya’s national guidelines included:

- Revising health workplans markedly reduced the average number of stock-out days of essential antibiotics and other commodities, noted as a key barrier to the management of sick young infants (SYIs) with possible serious bacterial infection (PSBI).
- Strengthening provider knowledge, skills, and confidence through on-the-job training and professional development led to an increase in the number of SYIs who were correctly classified, documented, and treated.
- Strengthening community linkages to primary health care facilities by training community health volunteers (CHVs) on the identification, prompt referral, and follow-up of SYIs, resulted in an increase in the number of SYIs with PSBI referred to primary health care facilities.

**Key Implications:**

- Institutionalizing the management of SYIs with PSBI where referral is not feasible through updated integrated management of newborn and childhood illness guidelines into subcounty and facility workplans and budgets ensured sustainability and increased uptake of services for SYIs.
- To improve guideline utilization, implementation of supply-side interventions at the facility level should be coupled with demand-side interventions at the community level, including empowering CHVs and caregivers with the right knowledge and skills to identify danger signs and refer SYIs promptly to health facilities.

## INTRODUCTION

Globally, 2.4 million children died in the first month of life in 2019. There are approximately 6,700 newborn deaths every day, amounting to 47% of all deaths in children aged younger than 5 years, up from 40% in 1990.^1^ Most of these deaths occurred in low- and middle-income countries. Bacterial infection is the third leading cause of neonatal mortality besides birth asphyxia and prematurity.^2^ Kenya’s continued neonatal mortality rate of 21 per 1,000 live births for the last 30 years implies that child mortality rates will likely remain high unless neonatal and young infant deaths are significantly reduced.^3^^,^^4^

Ideally, sick young infants (SYIs) aged 0–59 days with possible serious bacterial infection (PSBI) should be admitted to hospitals for inpatient care. However, many SYIs do not receive the ideal inpatient care due to caregiver refusal of admission and other factors, including long distances and poor transport infrastructure to and from referral sites.^5^ Two large randomized clinical trials commissioned by the World Health Organization (WHO), namely, the Simplified Antibiotic Therapy Trial (SATT) in Asia and the African Neonatal Sepsis Trial (AFRINEST) in Africa, showed that using a simplified antibiotic regimen to manage SYIs with PSBI provided at primary health care (PHC) facilities when referral is not possible was effective and could save lives. As a result, WHO released guidelines on managing PSBI in young infants aged 0–59 days at PHC facilities when referral is not feasible.^5^^,^^6^

Despite evidence that a simplified antibiotic regimen was efficacious and safe in randomized trials, there were concerns about its fidelity, feasibility, and acceptability in actual program implementation settings. Therefore, implementation research (IR) was used to expand uptake of the guidelines and simultaneously develop an understanding of these implementation concerns, including weak community-facility linkages, poor continuity of care-seeking, and lack of follow-up of SYIs by community health volunteers (CHVs). Based upon lessons from early IR conducted in 7 countries, WHO issued additional operational guidance in late 2017 to address some of the emerging implementation concerns in typical non-research health settings.^7^

Despite evidence that a simplified antibiotic regimen was effective and safe for sick young infants, there were concerns about its fidelity, feasibility, and acceptability in actual program implementation settings.

To design and evaluate an approach to introduce and sustain PSBI implementation in Kenya, the U.S. Agency for International Development (USAID) supported the Scaling up PSBI Guidelines in Kenya by Building Confidence in the Management of Sepsis in Young Infants, also known as the *Ponya Mtoto* (Swahili meaning “treat the baby”) project. We tested the feasibility, acceptability, sustainability, adoption, appropriateness, and fidelity of incorporating these new guidelines into routine maternal, newborn, and child health (MNCH) service delivery in Kenya (Box). We describe Kenya’s experience in institutionalizing the WHO guidelines for PSBI where referral is not feasible into the country’s national guidelines on integrated management of newborn and childhood illnesses (IMNCI).

BOX Definitions of Key Ponya Mtoto Implementation Outcome Measures

**Feasibility:** The extent to which the new integrated management of newborn and childhood illnesses (IMNCI)/possible serious bacterial infection (PSBI) guidelines can be successfully used within a given setting to manage sick young infants (SYIs) with PSBI where referral is not feasible.

**Acceptability:** The perception that management of SYIs using IMNCI/PSBI guidelines is acceptable or satisfactory to health providers and managers.

**Fidelity:** The degree to which the interventions were implemented according to the IMNCI/PSBI guidelines.

**Adoption:** The extent to which health providers used the IMNCI/PSBI guidelines to treat SYIs.

**Appropriateness:** The perceived fit of the intervention package to address the problem of high morbidity and mortality for young infants caused by PSBI. We specifically looked at the health provider’s and caregiver’s perceptions of the recommended PSBI treatment regimen.

**Sustainability:** The extent to which a newly implemented treatment is maintained or institutionalized within a service setting’s ongoing, stable operations.

## PONYA MTOTO PROJECT DESCRIPTION

The Ponya Mtoto project aimed to reduce young infant deaths caused by PSBI in 4 areas: (1) integrating new PSBI guidelines into IMNCI national training and management protocols, (2) demonstrating that new PSBI guidelines implementation was feasible, acceptable, and sustainable, (3) increasing utilization of quality PSBI care, and (4) changing national IMNCI guidelines. The project was led by Population Council and implemented in partnership with the Kenya Paediatric Research Consortium, Mount Kenya University, the Ministry of Health (MOH), and county health departments between October 2017 and June 2021.

### Project Setting

**FIGURE 1:**
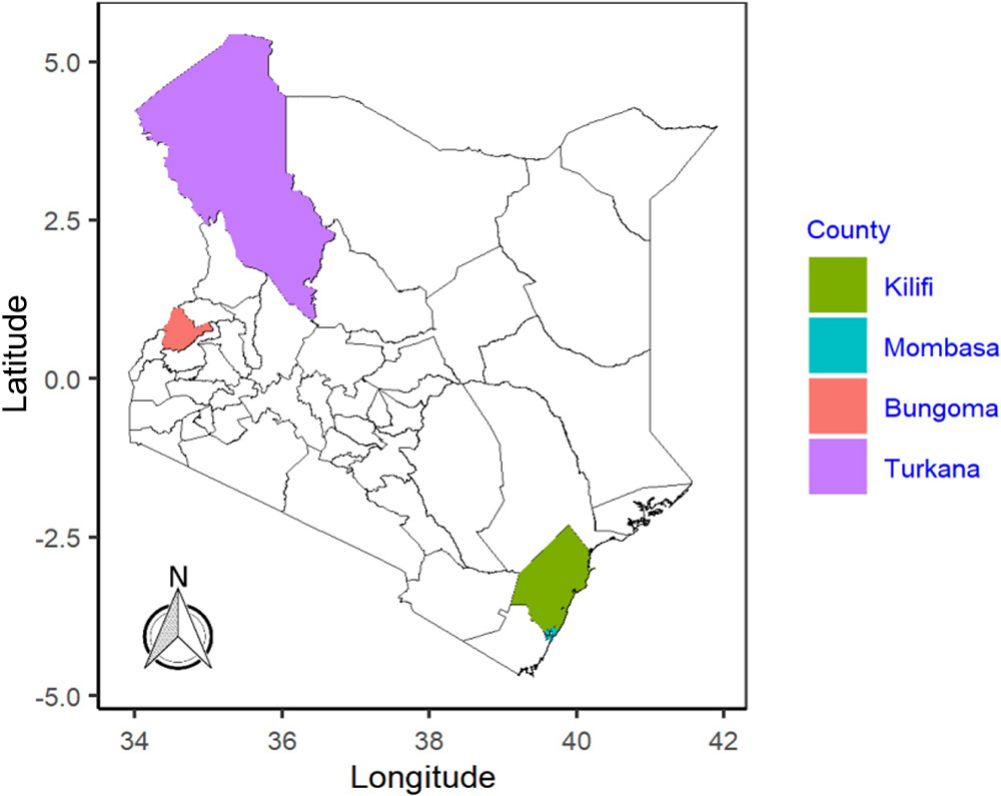
Map of Ponya Mtoto Project Counties, Kenya

With guidance from the MOH, Ponya Mtoto was implemented in 4 counties in Kenya that had higher infant and newborn mortality rates than the national mean (22/1000 live births): Bungoma (33/1000 live births), Kilifi (26/1000 live births), Mombasa (39/1000 live births), and Turkana (60/1000 live births) (Figure 1).^4^

These counties represented a mix of rural settings, urban informal settlement sites, poverty levels, diverse agroecological conditions, and nomadic pastoralist lifestyles that affect access to health care. Bungoma represents a rural agrarian population with cultural vulnerabilities; Kilifi represents a coastal mixed rural-urban setting with cultural vulnerabilities; Mombasa represents a coastal urban subpopulation with high poverty levels in informal settlements; and Turkana reflects a nomadic pastoralist lifestyle with cultural and geographical vulnerabilities. Two subcounties were selected from each county in consultation with the county health management teams (CHMTs).

Kenya’s health system comprises 6 tiers: national referral and teaching hospitals (level 6), county referral hospitals (level 5), subcounty hospitals (level 4), health centers (level 3), dispensaries (level 2), and community health structures (level 1). Hospitals are secondary and tertiary facilities that represent a higher level of care, while health centers and dispensaries (levels 2–3) are PHC facilities. CHVs are deployed at level 1 to conduct preventive and promotive health services in the community.

The 4 project counties have a total of 456 public sector health facilities comprising 28 hospitals, 56 health centers, and 372 dispensaries. In each county, the project team, in collaboration with respective CHMTs, identified 12 health facilities stratified by level of service delivery (2 hospitals, 4 health centers, and 6 dispensaries) as study sites based on the high volume of SYIs attended to each month. As such, a total of 48 health facilities served as the study sites (Table 1).

**TABLE 1.** Distribution of Ponya Mtoto Project Facility Types in 4 Counties in Kenya

**County and Subcounty**	**Hospitals**	**Health Centers**	**Dispensaries**
Bungoma			
Webuye West subcounty	2	1	3
Tongaren subcounty	1	3	2
Turkana			
Turkana Central subcounty	1	2	3
Turkana West subcounty	2	2	2
Mombasa			
Mvita subcounty	1	3	2
Changamwe-Jomvu subcounty	1	3	2
Kilifi			
Kilifi North subcounty	1	2	3
Kaloleni subcounty	1	3	2

Kenya’s MOH is responsible for policies and standards, revising guidelines, developing training materials and tools, and strengthening the capacities of CHMTs. Administratively, counties are subdivided into 5 to 17 subcounties at which level the subcounty health management teams (SCHMTs) operate. As a devolved function of the health system, health services are managed by county governments through the CHMTs and SCHMTs, who develop workplans, ensure availability of essential drugs and commodities, and provide supportive supervision to health facilities. Health providers are responsible for actual routine service provision to SYIs with PSBI (identification, classification, treatment, and documentation) at the various levels of care. CHVs’ roles include conducting home visits, identifying SYIs at the community level, and referring to PHC facilities within their catchment areas, as well as conducting follow-up visits of SYIs after initial treatment to ensure adherence to the treatment regimen.

### Project Activities

**FIGURE 2:**
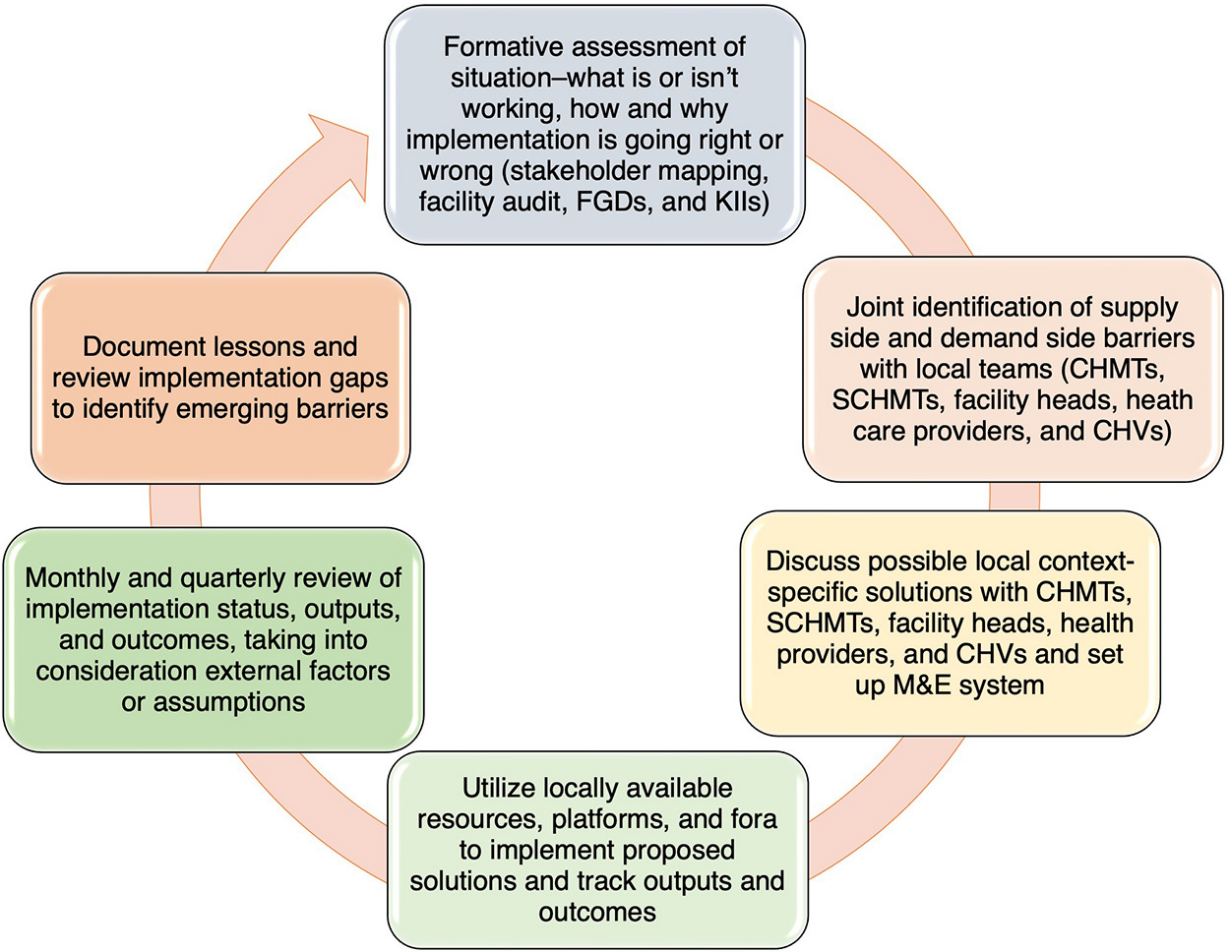
Ponya Mtoto Project Implementation Research Cycle Abbreviations: CHMT, county health management team; CHV, community health volunteer; FGD, focus group discussion; KII, key informant interview; M&E, monitoring and evaluation; SCHMT, subcounty health management team.

Ponya Mtoto activities were implemented as part of the IMNCI services at the community and PHC levels. Working with local teams to identify barriers and solutions to accessing health care for SYIs with PSBI, Ponya Mtoto used IR (Figure 2) to demonstrate how to adopt the WHO PSBI guidelines in the face of real-world implementation problems in real time.^8^^–^^10^

Pony Mtoto used IR to demonstrate how to adopt the WHO PSBI guidelines in the face of real-world implementation problems in real time.

The project team collected data during 3 periods: baseline or formative data collection (June–July 2018), endline data collection (December 2020–January 2021), and iterative monitoring (quarterly). The project team used both quantitative and qualitative data collection methods, including an organizational capacity assessment, focus group discussions, health facility audits, in-depth interviews with providers and CHVs, and case narratives with caregivers.

The formative assessment assisted in identifying the strengths and barriers in service provision to SYIs with PSBI where referral is not feasible. Monitoring data were used to gauge progress in implementing project interventions and tracking changes made in improving the management of SYIs with PSBI. The endline survey was conducted to assess the overall impact of project interventions, assess progress in improving management of SYIs with PSBI, document activities and procedures implemented that may have contributed to results or outcome measures, and document lessons.

## IMPLEMENTATION APPROACH AND HIGHLIGHTS OF FINDINGS

**FIGURE 3:**
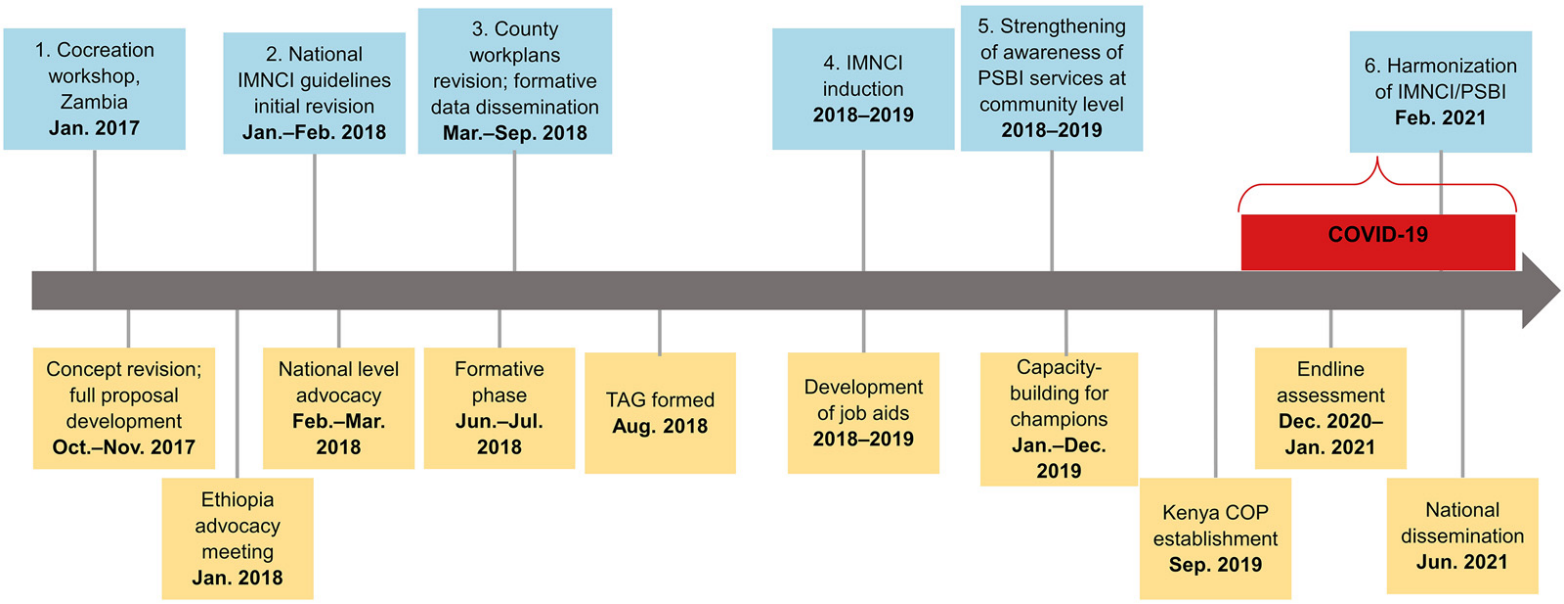
Timeline of Key Ponya Mtoto Project Milestones Abbreviations: CHV, community health volunteer; COP, community of practice; IMNCI, integrated management of newborn and child illnesses; PSBI, possible serious bacterial infection; TAG, technical advisory group.

We describe 6 activities that specifically contributed to the institutionalization of the management of SYIs in Kenya’s health system. These were: (1) participating in a prework cocreation workshop and developing a theory of change; (2) revising national IMNCI guidelines to incorporate the management of PSBI where referral is not feasible; (3) improving availability of essential commodities through revision of county health workplans; (4) strengthening provider confidence in the management of SYIs through on-the-job training; (5) strengthening awareness about PSBI services for SYIs at the community level; and (6) harmonizing national IMNCI guidelines to address content discrepancies on management of PSBI (Figure 3). We also describe the contribution of 2 cross-cutting themes, namely, using IR to guide programming and the role of functional quality improvement teams (QITs) in sustaining high-quality PSBI management.

### 1. Participation in a Prework Cocreation Workshop and Development of the Theory of Change

In January 2017, USAID organized a cocreation workshop in Zambia for country teams that had submitted expressions of interest as part of the USAID Broad Agency Announcement bidding process for research on operationalizing the WHO guidelines for PSBI where referral is not feasible. Workshop participants included multidisciplinary teams of researchers; MOH officials; and health providers from Bangladesh, Ethiopia, Kenya, Malawi, Myanmar, Nepal, Nigeria, and Pakistan. The workshop aimed to develop a shared appreciation of the WHO guidelines’ implementation challenges and opportunities using a human-centered design approach. Before the cocreation workshop, USAID shared a template that country teams used to obtain feedback from target populations, including health policymakers, providers, and caregivers of SYIs whose perspectives were synthesized and incorporated into the respective country concept notes.

**FIGURE 4:**
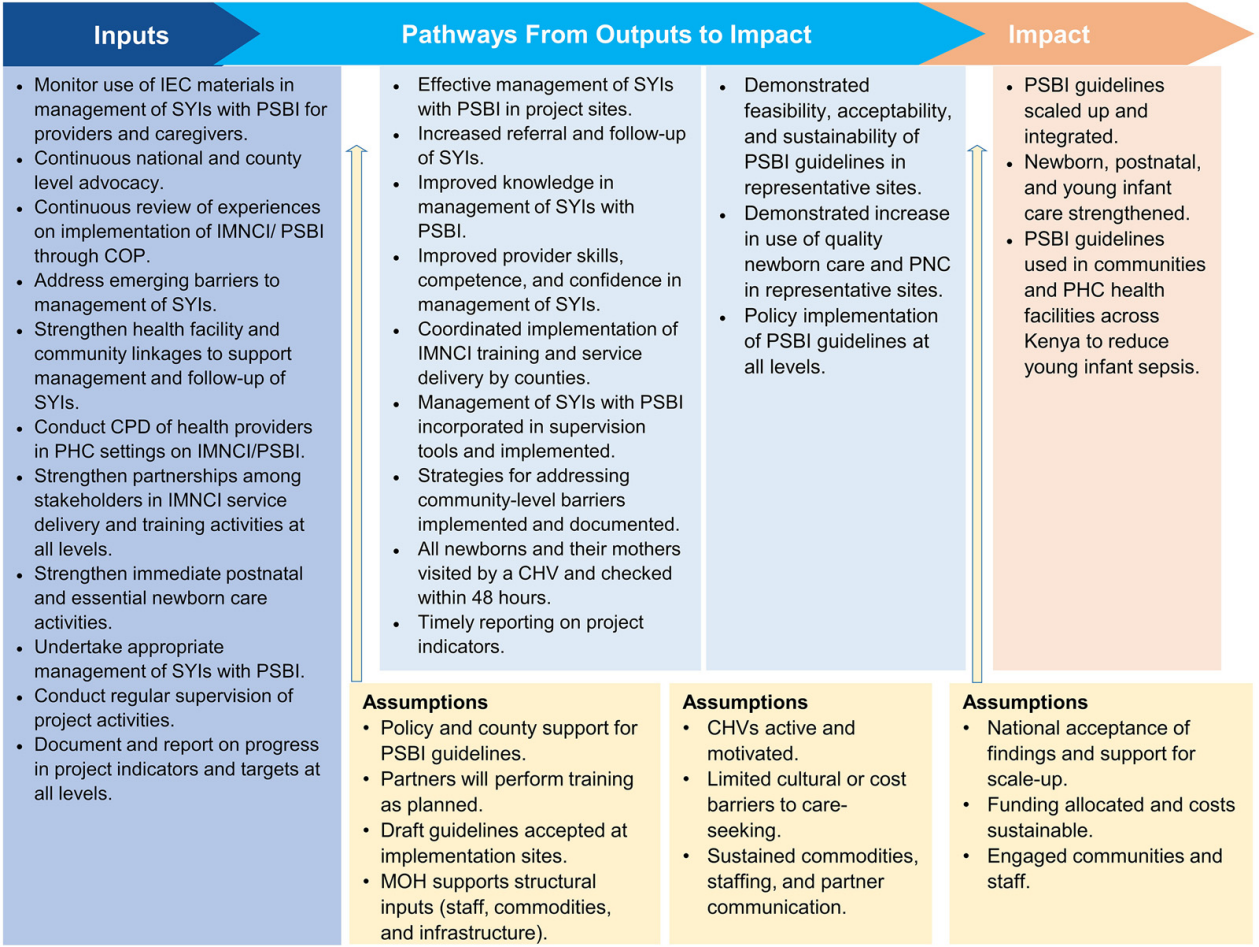
Ponya Mtoto Project’s Revised Theory of Change Abbreviations: CHV, community health volunteer; COP, community of practice; IEC, information, education, and communication; IMNCI, integrated management of newborn and child illnesses; MOH, Ministry of Health; PHC, primary health care; PSBI, possible several bacterial infection; SYI, sick young infant.

The concept notes formed the basis of developing the Ponya Mtoto project theory of change (TOC). The TOC outlined barriers that prevent SYIs from accessing prompt and timely care, the inputs required for the desired change, critical assumptions, and pathways from output to outcomes and impact. Based on experiences and challenges that emerged during the project implementation, the TOC was periodically revised (Figure 4). For example, on-the-job training, continuing professional development (CPD) of providers on IMNCI/PSBI, and strengthening of health facility–community linkages components were added in subsequent versions of the TOC, and the assumptions were revised accordingly.

Designing a responsive TOC to the needs of SYIs constituted the first step in institutionalizing the management of SYIs in routine health care service delivery. Following the precedent set in the prework cocreation workshop in Zambia, the Ponya Mtoto project team brought many stakeholders together, including health policymakers, providers, and caregivers of SYIs, to develop and revise the TOC over the project period. The use of human-centered design increased the ability of the Ponya Mtoto project team to address emerging implementation challenges because the approach considered the views and perspectives of caregivers of SYIs and their families, health providers, and county- and national-level health managers.^11^^–^^13^

Designing a responsive TOC to the needs of SYIs constituted the first step in institutionalizing the management of SYIs in routine health care service delivery.

Key drivers of the pathway from inputs through outputs to impact of the Ponya Mtoto TOC included: (1) learnings from a 2018 WHO PSBI Guidelines dissemination meeting in Ethiopia by the Kenyan delegation; (2) MOH leadership in mobilizing development partners to support the IMNCI guidelines revision to include PSBI where referral is not feasible; and (3) support by professional associations, such as Kenya Paediatric Research Consortium, which provided on-the-job training for health providers. An important assumption in the TOC was that other partners would have sufficient interest to scale up the management of SYIs with PSBI. At the end of the project period, the Clinton Health Access Initiative, UNICEF, PATH, and Bill & Melinda Gates Foundation had supported IMNCI/PSBI activities in 28 other counties beyond the initial 4 Ponya Mtoto project sites.

### 2. Initial Revision of National IMNCI Guidelines to Incorporate PSBI

Over the project period, the Kenya MOH spearheaded 2 revisions of the Kenya national IMNCI guidelines. The initial revision of the national IMNCI guidelines was brought about by broad lessons from the dissemination meeting organized by WHO in Ethiopia in January 2018 to share experiences from early PSBI IR in a few Asian and African countries. A key lesson learned from that meeting was that the management of SYIs with PSBI needs to be implemented within the country’s broader health system and integrated into the national child health policy and national IMNCI strategy/guidelines. The initial guidelines revision was undertaken in January–February 2018 at a forum organized by MOH officials who had recently returned from the WHO meeting in Ethiopia. Child health experts attending the forum included representatives from the MOH, professional health associations, health councils and boards, United Nations agencies (WHO and UNICEF), international organizations (Clinton Health Access Initiative, Population Council, Red Cross, PATH, among others), IMNCI master trainers, CHMTs, and health providers. The revision entailed incorporating the management of PSBI where referral is not feasible into the national IMNCI guidelines.

After this revision, the MOH, in collaboration with county and Ponya Mtoto teams, conducted on-the-job training of health providers on the revised IMNCI guidelines. Through supportive supervision visits, Ponya Mtoto and SCHMTs continued to monitor utilization of the revised IMNCI guidelines and document feedback from health providers (Table 2). These concerns and recommendations informed the subsequent harmonization of the IMNCI guidelines discussed in Section 6.

**TABLE 2.** Providers’ Concerns About 2018 IMNCI Revised Guidelines and Recommendations for Future Revision

**Category**	**Provider Concern**	**Recommendations**
**Day 2 follow-up**	Many caregivers bringing infants on day 2 to receive second dose of gentamycin tended to bypass consultation rooms and went directly to the injection rooms. Therefore, the SYIs missed the mandatory day 2 assessment. Partners felt that the day 2 assessment of the SYI was critical to ensure quality service provision.	Health providers to educate caregivers to return SYIs on day 2 for review before the second dose of gentamycin is administered.
**Day 4 follow-up**	Fewer caregivers returned their SYIs for review on day 4 because they felt their infants’ condition had improved, despite the guidelines requiring caregivers to bring back SYIs for review.	Health providers to educate caregivers on the need to return SYIs on day 4 for review to document whether the SYI’s condition is improving, has remained the same, or is deteriorating.
**Assessment**	The National IMNCI Chart Booklet lists 18 signs to be used in assessment of SYIs with PSBI, compared to 7 signs for assessment of SYIs in the PSBI section.	Revise the national IMNCI and PSBI materials to harmonize the signs and symptoms for assessment of SYIs.
**Classification**	The National IMNCI Chart Booklet lists 11 signs for critical illness, compared to 6 signs under critical illness in the PSBI section.	Revise the national IMNCI and PSBI materials to harmonize the signs and symptoms for classification of critical illness in infants presenting with PSBI.
**Fast breathing**	The National IMNCI Chart Booklet lumps together all infants aged 0–59 days with signs of fast breathing, compared to the PSBI section that makes a distinction between fast breathing in infants aged 0–6 days and infants aged 7–59 days.	Revise the national IMNCI and PSBI materials to distinguish fast breathing between infants aged 0–6 days and aged 7–59 days. Infants aged 0–6 days with fast breathing need to be referred for inpatient treatment. If referral is declined, treat as outpatient with oral amoxycillin daily for 7 days. Infants aged 7–59 days with fast breathing need to be treated in outpatient settings. Review on day 4 is mandatory for both groups.
**Temperature**	The National IMNCI Chart Booklet indicates temperature for fever as “Fever 37.5°C or above or feels hot,” compared to the PSBI section that indicate temperature for fever as “38°C” or more.	Revise the national IMNCI and PSBI materials to harmonize the value of temperature/fever.

Abbreviations: IMNCI, integrated management of newborn and childhood illnesses; PSBI, possible serious bacterial infection; SYI, sick young infant.

### 3. Strengthening of the Availability of Essential Commodities By Revising County Workplans

The formative assessment identified frequent stock-out of essential antibiotics and other supplies at the PHC level as a key barrier to optimal management of SYIs with PSBI. Between September and December 2018, Ponya Mtoto held forums with CHMTs and SCHMTs to disseminate and discuss findings from the formative assessment. Between August and October 2019, the project team shared county-specific technical briefs that presented results of the assessment of the health system and community readiness to scale up the PSBI treatment regimen for SYIs in the context of IMNCI. Ponya Mtoto used the forums and briefs to assist counties in revising their workplans to incorporate commodities for managing SYIs, training providers on revised IMNCI guidelines that incorporated PSBI activities, and strengthening supportive supervision, among other activities. While the project did not provide additional funding for procurement and distribution of essential PSBI commodities, it assisted CHMTs and SCHMTs to better use existing resources and leverage existing partnerships to ensure the availability of the commodities.

The project assisted CHMTs and SCHMTs to better use existing resources and leverage existing partnerships to ensure the availability of antibiotics and other commodities.

Ponya Mtoto also advocated for improvements in forecasting and quantifying essential commodities for managing SYIs, tracking trends in stock-outs of antibiotics and other supplies for managing SYIs, and using data for programming, with a view to institutionalizing and sustaining commodities supply and management. The project team, in conjunction with CHMTs and SCHMTs, held follow-up meetings to ensure that these challenges had been addressed and integrate the management of SYIs with PSBI where referral is not feasible, including essential antibiotics, into facility-level workplans and budget. As such, budgetary allocation for essential antibiotics and other supplies to health facilities was factored into annual county health budgets to ensure sustainability.

A review of monitoring data showed a marked reduction in the average number of stock-out days of amoxicillin dispersible tablets, a key antibiotic for managing SYIs with PSBI. The average number of stock-out days of amoxicillin dispersible tablets in dispensaries decreased from 204 days in quarter 4 of 2018 to 18 days in quarter 3 of 2020. The improvement in the availability of essential antibiotics can be attributed to advocacy by Ponya Mtoto in conjunction with other efforts to strengthen the county government’s capacity for commodity forecasting and quantification. Data from the endline survey showed increased integration of IMNCI/PSBI into routine health facility services and activities. For instance, more than 66% (32 of 48 facilities) had incorporated PSBI into their workplans and reports, and 71% (34 of 48 facilities) had incorporated PSBI drugs into their budgets, procurement plans, and requisitions.

It should be noted that another USAID-supported project, Afya-Ugavi, had trained key health personnel in commodities management in all counties in Kenya, which complemented the Ponya Mtoto project’s efforts that focused on essential PSBI commodities. Counties mainstreamed the procurement of the essential PSBI commodities within the county workplans and budgets to improve the likelihood of sustainability,^14^ as opposed to situations in which management of essential antibiotics relied on vertical donor-supported programs.

### 4. Strengthening of Provider Confidence in Management of SYIs with PSBI

Another barrier to effective management of SYIs with PSBI identified during the formative assessment was weak health provider knowledge, skills, and confidence in IMNCI/PSBI. To address this barrier, Ponya Mtoto worked with CHMTs and SCHMTs to strengthen the health providers’ capacity and confidence to identify and assess SYIs, classify them, and provide appropriate treatment. Capacity-strengthening activities comprised on-the-job training and on-site CPD sessions delivered by county pediatricians and county child health focal persons trained as IMNCI/PSBI champions. In PHC facilities, all providers working in the maternal-child health and outpatient departments were trained on IMNCI-PSBI. In hospitals, providers working in maternal-child health and outpatient departments, as well as those working in both the newborn units and pediatric wards, were also trained on IMNCI-PSBI. Training of health providers covered all tasks/activities in IMNCI, including triaging, assessment, classification and treatment, use of job aids, antibiotic regimen, and referral.

Another strategy adopted by the Ponya Mtoto project toward strengthening health provider capacity and confidence in management of SYIs was to establish the Kenya Community of Practice (KCOP). The KCOP was constituted in 2019 to bring together health professionals and institutions interested in the overall well-being of children, including SYIs with PSBI. The KCOP supported collaboration between governance levels (national teams versus county-level teams) and helped connect people who might not ordinarily interact. KCOP membership included international institutions, health policy and program managers, health providers, research institutions, and professional associations, among others. KCOP shared information through presentations and dashboards, webinars, local in-person meetings (e.g., facilities’ data-sharing fo-rums), email groups, online discussion forums, resource-sharing, professional champions, and video documentaries.

During the COVID-19 pandemic, the KCOP platform proved particularly helpful by enabling health providers and managers to interact virtually and learn best practices in managing SYIs. For instance, on the KCOP YouTube channel, Ponya Mtoto uploaded videos of real-time management of SYIs at various levels of care that professionals across the country could access. The videos presented different scenarios to assist health providers and managers in handling infant health. For example, they demonstrated how to identify SYIs and conduct referrals from the community to the health facility, how to manage SYIs with critical illness, and how to conduct continuous medical education sessions on management of PSBI, among others.

During the endline assessment, the project team assessed provider performance in the various IMNCI tasks undertaken in their respective facilities. The proportion of facilities whose providers had been trained on assessment of SYIs ranged from 86%–100%; classification and treatment from 94%–100%; use of PSBI job aids from 71%–86%; and on antibiotics regimen for managing SYIs from 77%–94%. Providers received job aids, including the IMNCI/PSBI management flowchart^15^ and PSBI Healthcare Provider Pamphlet. These efforts led to an increase in the number of SYIs who were correctly classified, documented, and treated over the project period. Project monitoring data also showed an improvement in the number of SYIs with PSBI correctly classified and documented as having critical illness, severe pneumonia, and pneumonia, from 157 in quarter 1 of 2019 to 517 in quarter 3 of 2020. Similar findings were reported in a study conducted in Ethiopia between 2018 and 2019 that showed that with good quality training and consistent supportive supervision, providers trained in IMNCI could correctly assess, classify, and treat SYIs in their respective facilities.^16^

To sustainably address weak health provider knowledge, skills, and confidence in IMNCI/PSBI, subcounty and health facility teams incorporated capacity-building activities into their workplans and budgets, as discussed earlier. The successful integration of IMNCI/PSBI activities into health facility workplans and budgets could be attributed to 2 factors. First, the planning of routine service delivery at the facility level was participatory, involving the Ponya Mtoto project team, MOH headquarters staff, and county and health facility teams. Unlike previous top-down approaches, this participatory approach entailed county- and facility-level staff identifying implementation gaps, working out strategies for addressing the gaps using resources at their disposal, monitoring implementation of the interventions, and periodically evaluating the success of the interventions. Second, implementation of IMNCI/PSBI activities was embedded in the public health system; planning, coordination, implementation, and resource allocation were undertaken within the existing country health structures. An Ethiopian study also highlighted similar facilitating factors for institutionalizing IMNCI/PSBI within the health system, including using a multidisciplinary approach and enhancing collaboration between different levels of governance.^17^

To sustainably address weak provider knowledge, skills, and confidence in IMNCI/PSBI, subcounty and health facility teams incorporated capacity-building activities into their workplans and budgets.

### 5. Strengthening Awareness on PSBI Services for SYIs at the Community Level

Weaknesses in community-facility linkages were identified during the formative assessment as a barrier to effective management of SYIs with PSBI across all sites. In response, Ponya Mtoto and the CHMTs provided an update training to CHVs on consistent home visits, identification of danger signs among SYIs, prompt referral of SYIs to health facilities for management, and follow-up of SYIs at the community level to ensure compliance with the treatment regimen. Monitoring data indicated an increase in the number of SYIs with PSBI referred by CHVs from the community to PHC facilities from 19 in quarter 1 of 2019 to 140 in quarter 3 of 2020. Overall, the number of CHV referrals rose from 21 in quarter 1 of 2019 to 172 in quarter 3 of 2020. Sustained follow-up by CHVs of SYIs who received initial treatment for PSBI contributed to an increase in the number of SYIs brought back for treatment by caregivers on Day 2 from 263 in quarter 1 of 2019 to 517 in quarter 3 of 2020. Similarly, the number of SYIs who returned to health facilities for follow-up on day 4 and day 8 increased from 0 in quarter 1 of 2019 to 266 and 140 in quarter 3 of 2020, respectively. It is worth noting that health facilities were able to use routine service utilization data to estimate demand for services and appropriately forecast the demand for commodities and services.

Ponya Mtoto also partnered with CHMTs to advocate for the provision of incentives by county governments to CHVs for their work. Advocacy was through meetings with county executive members for health in the respective counties. Subsequently, the county governments of Bungoma and Turkana enacted legislation to provide financial resources to strengthen the Community Health Strategy,^18^ including provision of a financial stipend for CHVs ranging from US$20 to US$30.^19^^,^^20^ The counties’ Community Health Services Act provides that the stipend will be sufficient to defray expenses reasonably incurred by CHVs in the discharge of their functions and will not be less than such sum as the County Community Health Services Committee will prescribe. This stipend is expected to ensure the sustainability of CHV service provision for SYIs and other health services because it is a part of county legislation.

Another barrier identified during the formative assessment was the prevalence of community sociocultural practices that negatively affected care-seeking for SYIs. Some of the common practices included delaying initiating breastfeeding while waiting for the naming ceremony, insisting on obtaining permission from the infant’s father or paternal grandmother before seeking care at a facility, and using traditional herbs to treat an SYI instead of going to a health facility. To address these barriers, the project worked with CHMTs, SCHMTs, and CHVs to develop and update job aids to strengthen awareness about PSBI services for SYIs. Ponya Mtoto developed a caregiver pamphlet (also translated into Swahili and Turkana languages), which CHVs disseminated in the community. The interventions at CHV and caregiver levels contributed to the steady increase in the number of SYIs referred from the community to health facilities.

In contrast to CHVs’ performance in Kenya, a study in India noted poor performance by community health workers on home visits and documentation of SYIs, despite having an incentives program.^21^ The study noted an almost complete absence of supportive supervision and postnatal home visits by community health workers. In Kenya, the project implemented activities through normal county health structures with supportive supervision of all health cadres, including CHVs, being embedded. In contrast, in India, the project established a technical support unit that did not supervise community health workers. A key lesson learned from the Ponya Mtoto project is that teaching CHVs and caregivers about signs and symptoms led to prompt identification and referral of SYIs to health facilities for care.

### 6. Harmonization of National IMNCI Guidelines to Address Content Discrepancies

In February 2021, the MOH undertook the harmonization of the IMNCI guidelines based on feedback and recommendations by health providers and managers and implementation experience. The harmonization effort also drew upon new evidence compiled by WHO upon further analysis of the AFRINEST trial data, as well as data from the Ponya Mtoto endline survey conducted in December 2020–January 2021 (Table 3).

**TABLE 3.** Harmonization of Kenya IMNCI-PSBI Guidelines

**Previous Content in Kenya IMNCI Guidelines**	**Harmonized Content in Line With WHO Recommendations**
**Classification** 18 signs to guide classification for PSBI/serious disease on page 32 in Kenya IMNCI Chart Booklet	Signs and symptoms harmonized as follows: Convulsions or convulsing now or Not able to feed at all or not feeding well or Fast breathing (60 breaths/minute or more in infants aged <7 days) or Serious chest indrawing or High body temperature (38°C or above) or Low body temperature (less than 35.5°C) or Movement only when stimulated or No movements at all
**Fast breathing** All infants aged 0–59 days lumped together (pages 32 and 37) in the Kenya IMNCI Chart Booklet	Revised Kenya IMNCI chart booklet distinguishes between fast breathing in infants aged 0–6 days and aged 7–59 days. Infant aged 0–6 days with fast breathing needs to be referred for inpatient treatment. If referral is declined, treat as outpatient with oral amoxicillin daily for 7 days. Infant aged 7–59 days treat in outpatient settings. Review on day 4 is mandatory for both groups.
**Critical Illness** 11 signs for critical illness on page 37 of Kenya IMNCI Chart Booklet	Signs for SYIs with critical illness (has any 1 of the following): Convulsions or convulsing now Not able to feed at all No movement on stimulation Weight <1500 g

Abbreviations: IMNCI, integrated management of newborn and childhood illnesses; PSBI, possible serious bacterial infection; SYI, sick young infant; WHO, World Health Organization.

Evidence from project monitoring data showed an improvement in the number of SYIs with PSBI correctly identified using the revised IMNCI job aids in PHC facilities, from 263 in quarter 1 of 2019 to 517 in quarter 3 of 2020. Similarly, there was an improvement in the number of SYIs with PSBI correctly classified and documented as having critical illness, severe pneumonia, and pneumonia, from 157 in quarter 1 of 2019 to 517 in quarter 3 of 2020.

Ponya Mtoto also worked with the MOH to update the register for children aged younger than 5 years and reporting tools by adding some of the PSBI indicators into the DHIS2. The additional PSBI indicators included: causes and number of neonatal deaths (prematurity, sepsis, and birth asphyxia); number of referrals to health facilities from the community by CHVs; children aged younger than 5 years presenting with pneumonia treated with amoxicillin dispersible tablets; children with pneumonia; and children with severe pneumonia.

A key lesson learned was that government ownership and leadership were critical in driving the process of revising and harmonizing the national guidelines on IMNCI and PSBI where referral is not feasible, as well as in incorporating the additional PSBI indicators into the DHIS2 and registers. The MOH was engaged from the beginning at the 2018 meeting in Ethiopia and convened the meeting that undertook the initial revision of the IMNCI and PSBI guidelines in Kenya. The MOH took leadership in disseminating the revised register and reporting tools to the rest of the 43 counties that were not part of the project. Further, the MOH worked with CHMTs and SCHMTs and took leadership in supporting counties to integrate revised IMNCI activities into county workplans and budgets, including budgetary allocation for essential commodities for managing SYIs with PSBI. The MOH also supported county teams in spearheading CPD sessions on IMNCI including PSBI where referral is not feasible.

Government ownership and leadership were critical in driving the process of revising and harmonizing the national guidelines on IMNCI and PSBI and in incorporating the additional PSBI indicators into the DHIS2 and registers.

The role of government leadership has also been highlighted by other studies. For instance, a study undertaken in Nigeria reported that implementation of revised guidelines at scale required government commitment to strengthen the health system.^22^ To increase utilization of the revised national IMNCI guidelines, Ponya Mtoto translated some of the job aids into local languages, Swahili and Turkana. Evidence shows that interventions that seek to engage and empower patients, caregivers, and families can promote better care, including improved outcomes,^23^ if information and educational methods are tailored for each respective audience.^24^

## CROSS-CUTTING THEMES

During the formative phase of the project, CHMTs and SCHMTs pointed out the need to improve the provision of quality services within their respective health facilities and were also eager to learn how to use IR to identify emerging challenges and document solutions.

### Strengthening QITs to Sustain Quality of Care for SYIs

During the formative assessment, wide variations were noted in terms of the presence and functionality of QITs. Therefore, Ponya Mtoto and the CHMTs assessed the QIT functionality parameters, as provided in the Kenya Quality Model for Health guidelines.^25^ Ponya Mtoto advocated to revive inactive QITs, to incorporate IMNCI and PSBI in quality improvement activities, to hold regular QIT meetings, to develop and implement quality improvement action plans and capacity strengthening, and monitored the process of ensuring QITs were functional.

The endline survey results showed that 88% of the facilities maintained functional QITs, and 69% of facilities had developed quality improvement plans. We also assessed whether facility QITs had held at least 1 meeting in the quarter preceding endline data collection. Five of 10 hospitals, 13 of 18 health centers, and 13 of 20 dispensaries indicated they held 1 or more QIT meetings. The results from monitoring data showed that facilities with functional QITs had improved client flow, classification, and documentation of SYIs and had reduced the number of commodity stock-out days.

### Using IR to Guide Programming

The Ponya Mtoto project used IR as a tool to guide programming throughout the project period. From our perspective, IR is the implementation of proven interventions in the real world, accounting for contextual factors that affect the processes and outcomes. It involves analyzing the decisions in real time and engaging stakeholders and implementers in identifying the problems and solutions. Findings from the monitoring and endline data showed that CHMTs used the iterative IR sessions to plan and implement activities, adjust interventions, address emerging challenges, and apply what they were learning to improve service delivery for SYIs.^26^ This translated into effective and efficient adaptive learning by end-users of the new guidelines. County-level staff appreciated the IR approach because it enabled them to identify barriers and work out site-specific solutions.

*Initially, we had shortage of staff and sick children were not being assessed properly because we had not been trained. Sick children were also mixed with other patients on the queue. We discussed among ourselves and introduced triage system. Some of our staff were trained through [on-the-job] on triaging. We started triaging children focusing on those with serious problems. We also discussed the issue of staff shortage and requested our managers to post more clinicians. Currently, we have a good number of clinical staff, and we can even rotate freely without getting burn-out while treating sick young infants.* —Provider, Kilifi County

County-level staff appreciated the IR approach because it enabled them to identify barriers and work out site-specific solutions.

In terms of overall impact, the Ponya Mtoto project contributed to improvement in a number of implementation outcomes. For instance, regarding feasibility, there was an increase in the number of SYIs with PSBI who were correctly identified in PHC facilities, from 263 in quarter 1 of 2019 to 517 in quarter 3 of 2020. In addition, an average of 88% of providers had received training on key IMNCI/PSBI topics through CPD: assessment of SYIs, classification and treatment, use of PSBI job aids, and types of antibiotic regimen for managing SYIs. Similarly, 71% of facilities had incorporated PSBI drugs in the facility budgets and procurement plans, making it possible for these commodities to be purchased by the county governments.

The Ponya Mtoto project findings are similar to results documented in studies in India and Nigeria that showed that implementation of the WHO guidelines on management of SYIs with PSBI when referral is not feasible can contribute to saving infant lives.^21^^,^^27^

## OVERALL CHALLENGES

Despite the achievements highlighted, the Ponya Mtoto project experienced the following challenges.

- Some sociocultural practices that had a negative impact on health-seeking behavior for SYIs persisted.
- Weaknesses in community-facility linkages, including long distances traveled by caregivers to health facilities, undermined referral and follow-up of SYIs at the community level.
- The COVID-19 pandemic and accompanying containment measures made it difficult to continue normal operations, including conducting Ponya Mtoto activities (e.g., quarterly monitoring and face-to-face data collection), thereby causing delays in the pace of project implementation.
- Prolonged industrial action by health care workers in Kenya calling for higher pay and provision of COVID-19 personal protective equipment between November 2020 and February 2021 interrupted service provision in all public health facilities, including the Ponya Mtoto sites.
- Inadequate time for implementing project activities made durable institutionalization and sustainability difficult to be achieved and understood.

## LESSONS LEARNED

Timely case identification and initiation of suitable antibiotic treatment at the PHC level is necessary, especially given the high rate of neonatal mortality in Kenya coupled with high refusal rates by caregivers for inpatient management of SYIs with PSBI. This implies that the use of a simplified antibiotic regimen in managing SYIs will continue to be relevant in the Kenyan context in the future. The overall lessons learned from the implementation of the project can be appreciated at the micro-, meso-, and macrolevels.

### Microlevel (Caregivers, CHVs, and Providers)

Caregivers found the treatment regimen for managing SYIs with PSBI to be acceptable, as new guidelines allowed them to attend to other domestic chores, including caring for other children, that would otherwise have been left with neighbors or friends if the SYIs had been referred and admitted for inpatient care. In addition, caregivers cited the important role CHVs played during at-home SYI follow-up visits to check on their conditions and to remind the caregivers to complete the treatment schedules.

Capacity-building of CHVs on the revised IMNCI guidelines that incorporated PSBI where referral is not feasible was critical in strengthening caregiver awareness of danger signs and facilitating prompt identification and referral of SYIs to health facilities for treatment.

Similarly, the majority of health providers found the simplified PSBI treatment regimen more convenient for caregivers as they were able to follow instructions and adhere to treatment schedules. The providers also found the simplified antibiotic regimen easy to administer and store at the facility.

### Mesolevel (Health Facilities and County)

The majority of health facilities institutionalized SYI management with the use of revised national IMNCI/PSBI guidelines, and this led to improved uptake of services for SYIs where referral is not feasible. Provider capacity-strengthening by Ponya Mtoto and CHMTs through a blended approach that combined on-the-job training, CPD sessions, and online platforms was an important approach in ensuring that knowledge generation and skills acquisition were sustained over time. The use of the revised IMNCI guidelines was found to be feasible, acceptable, and sustainable in the Kenyan community and PHC settings. IR helped CHMTs, SCHMTs, and facility-level teams to plan and track implementation; address emerging implementation challenges; and generate timely, context-specific/relevant learnings to inform their health service provision.

### Macrolevel (National Level Policy and Strategic Activities)

Four lessons stand out at the national level.

1. Kenya’s experience in revising the national IMNCI guidelines has shown that institutionalizing the management of SYIs with PSBI where referral is not feasible is possible when the specificities of the national health system and the stakeholders at the micro-, meso-, and macrolevels are considered and involved.
2. Institutionalizing the revised IMNCI guidelines, including health provider training, commodity supply chain, and health management information systems into subcounty and facility workplans and budgets, had the potential to ensure sustainability of service provision for SYIs.
3. Embedding IR into local health systems helped ensure stakeholder engagement, address the concerns of implementers and caregivers in a timely manner, and facilitate improvements in service delivery with minimal investments.
4. Alignment between the Kenya IMNCI/PSBI guidelines and WHO PSBI guidelines has helped providers in Kenya to use the IMNCI/PSBI Chart Booklet to distinguish the management of fast breathing between infants aged 0–6 days and 7–59 days. Previously, all infants aged 0–59 days with fast breathing had been lumped together in the Kenya IMNCI Chart Booklet. Currently, infants aged 0–6 days with fast breathing need to be referred for inpatient treatment, and infants aged 7–59 days presenting with fast breathing only can be managed effectively on an outpatient basis in PHC facilities.

## CONCLUSION

Using an IR approach to introduce new WHO guidelines on PSBI where referral is not feasible into Kenya’s health care service was critical to fostering engagement of a diverse range of stakeholders from caregivers of SYIs to national policymakers, monitoring provider skills and confidence-building, strengthening provision of key commodities for managing SYIs with PSBI, and sustaining community-facility linkages. Embedding the iterative review process within the county implementation plans strengthened the uptake of the PSBI guidelines and the use of services for SYIs.

Countries implementing PSBI where referral is not feasible in the context of IMNCI could benefit from practical implementation details and lessons learned from this study. A follow-up survey after 3 to 5 years would be helpful in assessing the benefits and coverage of institutionalizing the management of PSBI where referral is not feasible across counties in Kenya and documenting emerging challenges.

## Notes

**Peer Reviewed**

**First published online:** April 13, 2023.
